# Performance of GPT-4o and o1-Pro on United Kingdom Medical Licensing Assessment-style items: a comparative study

**DOI:** 10.3352/jeehp.2025.22.30

**Published:** 2025-10-10

**Authors:** Behrad Vakili, Aadam Ahmad, Mahsa Zolfaghari

**Affiliations:** 1 Department of Ophthalmology, St Thomas’ Hospital, Guy’s and St Thomas’ NHS Foundation Trust, London, UK; 2 Department of Trauma and Orthopaedics Surgery, Buckinghamshire Healthcare NHS Trust, Aylesbury, UK; 3 Department of Computer Science, University of Staffordshire London, London, UK; The Catholic University of Korea, Korea

**Keywords:** Artificial intelligence, Machine learning, Medical education, Medical licensure, United Kingdom

## Abstract

**Purpose:**

Large language models (LLMs) such as ChatGPT, and their potential to support autonomous learning for licensing exams like the UK Medical Licensing Assessment (UKMLA), are of growing interest. However, empirical evaluations of artificial intelligence (AI) performance against the UKMLA standard remain limited.

**Methods:**

We evaluated the performance of 2 recent ChatGPT versions, GPT-4o and o1-Pro, on a curated set of 374 UKMLA-style single-best-answer items spanning diverse medical specialties. Statistical comparisons using McNemar’s test assessed the significance of differences between the 2 models. Specialties were analyzed to identify domain-specific variation. In addition, 20 image-based items were evaluated.

**Results:**

GPT-4o achieved an accuracy of 88.8%, while o1-Pro achieved 93.0%. McNemar’s test revealed a statistically significant difference in favor of o1-Pro. Across specialties, both models demonstrated excellent performance in surgery, psychiatry, and infectious diseases. Notable differences arose in dermatology, respiratory medicine, and imaging, where o1-Pro consistently outperformed GPT-4o. Nevertheless, isolated weaknesses in general practice were observed. The analysis of image-based items showed 75% accuracy for GPT-4o and 90% for o1-Pro (P=0.25).

**Conclusion:**

ChatGPT shows strong potential as an adjunct learning tool for UKMLA preparation, with both models achieving scores above the calculated pass mark. This underscores the promise of advanced AI models in medical education. However, specialty-specific inconsistencies suggest AI tools should complement, rather than replace, traditional study methods.

## Graphical abstract

[Fig f4-jeehp-22-30]

## Introduction

### Background/rationale

The recent development of sophisticated artificial intelligence (AI) systems is revolutionizing medical education [1]. These systems function by processing large volumes of data, detecting patterns, and applying algorithms to make predictions, execute decisions, or perform actions [2,3]. AI’s growing impact in healthcare is evident, particularly in radiological imaging and histopathological analysis, where it assists in improving diagnostic accuracy and efficiency [4]. One rapidly expanding area of AI is the application of large language models (LLMs), such as ChatGPT. A growing body of literature now examines LLMs in medical education, with an increasing focus on their educational implications rather than purely technical performance. This includes their potential role in shaping curriculum design and influencing assessment strategies. These models are capable of engaging in detailed dialogue, explaining complex concepts, and even generating plausible clinical reasoning [5].

Early studies demonstrated that ChatGPT (GPT-3.5) could perform close to the passing mark for the United States Medical Licensing Examination (USMLE) [6]. In early 2023, GPT-3.5 achieved 55%–60% on USMLE items, approximating the around 60% passing threshold. GPT-4, however, attained much higher scores, averaging 86% on USMLE items [7]. These findings highlighted both the information-processing capabilities of ChatGPT and its potential as a valuable tool to support medical education and clinical practice. While previous work has largely focused on international assessments such as the USMLE, evaluations of LLMs against the United Kingdom Medical Licensing Assessment (UKMLA) remain scarce. This represents a crucial gap, given the UKMLA’s central role in medical education and assessment in the United Kingdom. Examining LLM performance in this context not only establishes a foundation for technical benchmarking but also provides insight into future implications for education and assessment. Nonetheless, AI has yet to be fully integrated into mainstream curricula and remains largely experimental in most training programs, due to limited research and regulation in this area [8].

The UKMLA is the standardized examination all UK medical students must pass to graduate. Its Applied Knowledge Test is a single-best-answer (SBA) examination comprising 200 items, without negative marking, covering a wide content map of clinical specialties and professional knowledge areas. Empirical analyses of LLMs on the UKMLA are limited, likely because the exam is relatively new.

### Objectives

We aim to evaluate the performance of 2 LLMs, GPT-4o and o1-Pro, neither of which has previously been studied in this context, on UKMLA mock items. We also seek to explore their potential in supporting image-based clinical analysis, an area which, to our knowledge, has not yet been examined in relation to the UKMLA.

## Methods

### Ethics statement

This study did not involve human participants, identifiable personal data, or animal subjects. Therefore, review and approval by the institutional review board were not required. The research consisted solely of secondary analysis of anonymized educational assessment material from a UK item bank, Geeky Medics (https://geekymedics.com/). Official permission to use the item bank materials was obtained from the copyright holders.

### Study design

This was a descriptive, comparative analysis evaluating the performance of 2 OpenAI models (GPT-4o and o1-Pro) on a curated dataset of SBA items modelled after the UKMLA. A paired design was employed, with both models answering an identical set of items, enabling direct comparison.

### Setting

The study was conducted using OpenAI’s ChatGPT interface. Each item was entered individually into both AI models (GPT-4o and o1-Pro), without external tool integration or contextual memory. To minimize memory bias, each item was entered into a new chat window. Items were collected from Geeky Medics between 1 April 2025 and 30 May 2025.

### Participants

Two advanced ChatGPT models, GPT-4o and o1-Pro, served as the “participants.” All items were screened for suitability by 2 medical professionals.

### Variables

The primary outcome was accuracy, defined as the percentage of items answered correctly by each model. Secondary outcomes included performance stratified by medical specialty and analysis of discordant responses between models. Items were categorized by specialty according to the official UKMLA content map.

### Data sources/measurement

A total of 394 UKMLA-style SBA items were screened from a licensed item bank. Permission for academic, non-commercial research use was granted by the copyright holder. Of these, 374 text-only items were included (Dataset 1). Each model’s responses were recorded in Microsoft Excel and compared with the official answers. Twenty image-based items were analyzed separately (Dataset 2). Items covered clinical photographs (e.g., dermatology, ophthalmology), radiological imaging (X-ray, computed tomography, magnetic resonance imaging), electrocardiography, and histopathology slides. Two resident doctors independently screened all items for suitability, applying inclusion criteria of a clear SBA format (single stem, 5 options, 1 best answer) and relevance to the UKMLA syllabus. Items with incomplete stems, duplicates, or requiring interactive/multimedia features not replicable in ChatGPT were excluded. Discrepancies were resolved by consensus; no third-party adjudication was required. Image-based material was presented at 904×740 pixels along with stem text where applicable. Images were uploaded directly into ChatGPT, and no feedback or correctness indication was given. Model parameters were left at ChatGPT defaults (temperature=1.0, top_p=1.0, max tokens=default). Each question was entered verbatim with a standardized prompt: “Please answer the following UKMLA-style single best answer question by selecting 1 option (A–E). Provide only the letter of the option you believe is correct.” For consistency, no follow-up clarifications or feedback were provided.

### Bias

To minimize memory bias, each item was tested in a new chat window. Neither model had access to external tools such as web browsing, and no correctness feedback was provided.

### Study size

A total of 374 text-based and 20 image-based items were arbitrarily selected after screening for suitability. No formal sample size calculation was performed.

### Statistical methods

Descriptive statistics were used to analyze the data with IBM SPSS ver. 28.0 (IBM Corp.), and results were tabulated and graphed in Microsoft Excel 2021 (Microsoft Corp.). To assess whether o1-Pro’s accuracy differed significantly from GPT-4o’s, McNemar’s test for paired binary outcomes (correct/incorrect) was applied. This test specifically evaluates discordant pairs—here, items answered correctly by one model and incorrectly by the other. A 2-sided P-value <0.05 was considered statistically significant. All analyses were conducted using Python (Pandas for data handling) and IBM SPSS ver. 28.0 for McNemar’s test. Codes found in Supplement 1.

## Results

### Overall performance

**Fig. 1.**
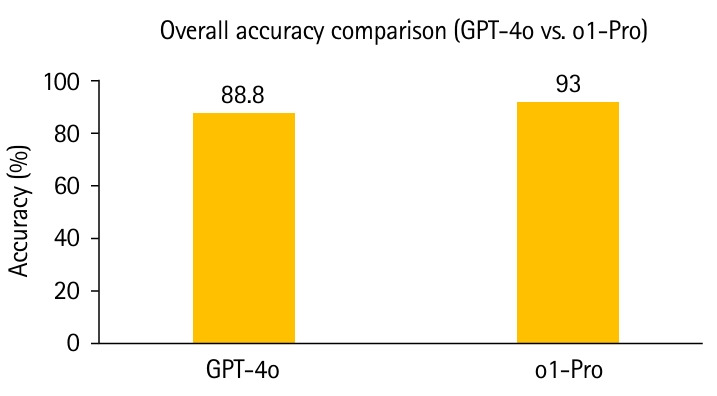
Overall performance of GPT-4o vs. o1-Pro on 374 UK Medical Licensing Assessment-style single-best-answer questions.

Out of 374 UKMLA-style items, GPT-4o answered 332 correctly (88.8% accuracy), while o1-Pro answered 348 correctly (93.0%). Both models comfortably exceeded the expected pass threshold, which was set at 57.5% based on the January 2025 UKMLA sitting. Fig. 1 illustrates the overall accuracy of each model. Notably, o1-Pro’s accuracy was approximately 4.2 percentage points higher than GPT-4o’s. At the item level, o1-Pro correctly answered 20 items that GPT-4o missed, whereas GPT-4o answered 4 items that o1-Pro missed, resulting in 24 discordant pairs. McNemar’s exact test confirmed that o1-Pro’s advantage was statistically significant (P=0.0015). As a measure of effect size, the odds ratio (OR) for discordant pairs was 5.00 (95% confidence interval [CI], 1.71–14.63), indicating substantially greater odds that an item missed by GPT-4o would be answered correctly by o1-Pro than vice versa.

### Performance by specialty

Table 1 provides a breakdown of results across medical specialties. The most pronounced difference was observed in general practice (GP) items, which often integrate multi-system knowledge and primary care scenarios. GPT-4o answered 10 of 14 GP items correctly (71.4%), while o1-Pro answered 12 (85.7%). This 14.3-point difference suggests that the updated model handled generalist items more effectively.

**Fig. 2.**
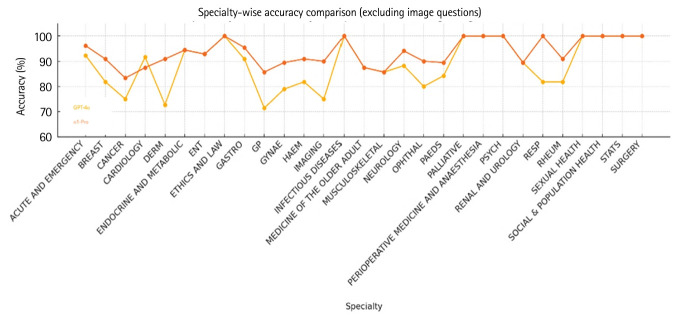
Specialty-wise accuracy of GPT-4o (orange) and o1-Pro (red).

Interestingly, both models showed relative weaknesses in GP, with lower accuracy compared to other specialties. Nevertheless, their performance in all domains remained above the pass threshold. Fig. 2 visualizes the accuracy of GPT-4o and o1-Pro across specialties, highlighting areas where o1-Pro (red) outperformed GPT-4o (orange).

### Image-based items

**Fig. 3.**
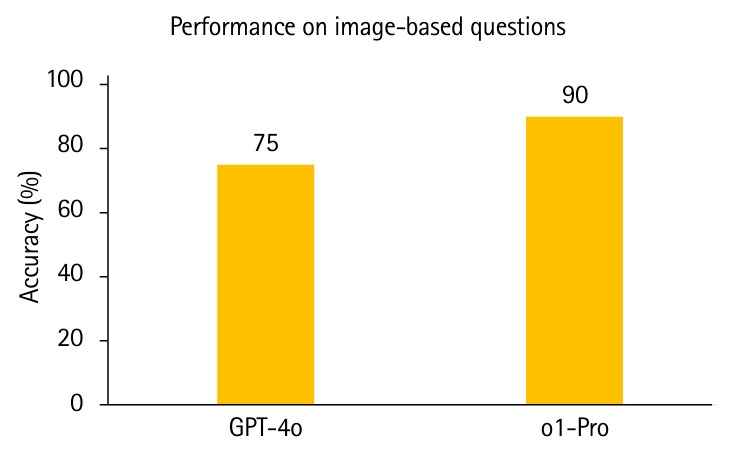
Comparison of GPT-4o and o1-Pro accuracy on image-based UK Medical Licensing Assessment questions. o1-Pro achieved a higher raw accuracy (90.0%) compared to GPT-4o (75.0%).

A separate analysis was conducted on the subset of image-based items (n=20). o1-Pro achieved an accuracy of 90.0%, while GPT-4o reached 75.0% (Fig. 3). The contingency table showed that both models answered 15 items correctly, with o1-Pro uniquely answering an additional 3 items that GPT-4o missed. McNemar’s test indicated that this difference was not statistically significant (P=0.25). To quantify the effect, the discordant pair odds ratio was large but imprecisely estimated due to zero counts in 1 cell. Applying a standard 0.5 continuity correction, the OR was 7.00 with a wide 95% CI of 0.36–135.52. Given the small sample (n=20) and the resulting low statistical power, this null result and wide CI warrant cautious interpretation. Accordingly, we present the image-based analysis as exploratory and hypothesis-generating rather than confirmatory.

## Discussion

### Key Results

Out of 374 UKMLA-style items, GPT-4o scored 88.8% and o1-Pro 93.0%, both well above the 57.5% pass mark. o1-Pro’s 4.2-point advantage was statistically significant (OR, 5.00; 95% CI, 1.71–14.63; P=0.0015). Specialty analysis highlighted o1-Pro’s relative strength in GP. On the 20 image-based items, o1-Pro achieved 90.0% compared with GPT-4o’s 75.0%, although the difference was not statistically significant.

### Interpretation/comparison with previous studies

This study investigated the performance of GPT-4o and o1-Pro on the UKMLA, representing the first evaluation of these models within this context. Previous studies of GPT-4 (released in 2023) on the UKMLA were limited by smaller sample sizes and a lack of analysis of image-based SBA items [4,9].

By situating our evaluation within the UKMLA’s assessment structure, particularly the Applied Knowledge Test, which relies on large numbers of SBA items including both text and images, our findings contribute directly to educational measurement. The use of accuracy relative to the published pass mark, specialty-level analysis mapped to the exam blueprint, and effect sizes from McNemar’s test together provide insight into how LLMs perform against the constructs the UKMLA is designed to assess.

The results of 88.8% (GPT-4o) and 93.0% (o1-Pro) indicate that both models achieved performance levels exceeding the expected standards of medical students. GPT-4o already surpassed the estimated minimum UKMLA standard, while o1-Pro even rivalled top-decile human performance. Importantly, we emphasized magnitude-based interpretation using effect sizes (ORs and 95% CIs) rather than relying solely on significance testing. These findings align with prior studies showing GPT-4’s strong performance on other licensing and specialty examinations, including the USMLE [7], Thailand’s National Medical Licensing Examination [10], the Peruvian National Medical Examination [11], and specialty board assessments such as ophthalmology and dermatology [12,13]. By contrast, GPT-3.5 (2022) achieved only 41%–60% on USMLE items, whereas GPT-4 (2023) improved to the mid-80s [7]. However, not all results have been uniformly positive; for example, GPT-4o scored 52% on the Korean Medical Licensing Examination, lower than GPT-3o’s 58% [14].

Our direct comparison of GPT-4o and o1-Pro suggests that fine-tuning and alignment updates can reduce errors and create more effective revision tools, with o1-Pro correctly answering 16 items missed by GPT-4o.

To date, some studies have investigated the ability of ChatGPT to recognize and analyze specific images. Image-based items from the Orthopaedic In-Training Examination were input into GPT-4o. Results indicated that GPT-4o produced insufficient performance for reliable imaging diagnosis, and further studies were recommended. GPT-4o and o1-Pro show important improvements over the earlier GPT-4 in multimodal tasks such as image analysis. Both GPT-4o and o1-Pro have high visual fidelity, with the latter excelling particularly in clinical visual tasks in radiology.

Our findings revealed a noteworthy performance: GPT-4o correctly answered 15 out of 20 image-based items (75%), while o1-Pro achieved an even higher accuracy with 18 out of 20 correct responses (90%). This suggests that LLMs may possess diagnostic capabilities and visual interpretation skills sufficient to be used as supplementary tools alongside textbooks for medical students, especially in imaging specialties such as radiology and dermatology.

The robust performance of ChatGPT on UKMLA-style items suggests that these models can act as high-quality supplemental tutors. Students can use ChatGPT to quiz themselves on practice items and immediately receive detailed explanations. Prior studies have noted that ChatGPT’s answers often contain logical rationales and relevant details from the item stem [15]. Interacting with ChatGPT in study mode could reinforce knowledge through elaboration. Some researchers have argued that if an AI can answer an item with a coherent rationale, it challenges educators to design questions that assess deeper understanding [16]. For now, students can benefit from existing item banks enhanced by AI. Our findings specifically highlight that both GPT-4o and o1-Pro would comfortably pass the UKMLA. Thus, a student could conceivably learn by reviewing topics with ChatGPT until the AI selects the correct answer, a form of mastery learning supported by AI [17]. This might be particularly useful in resource-limited settings or for re-sitting candidates without access to tutors. The concept of a smarter, more sustainable medical education emerges here: AI tutors such as ChatGPT can scale individualized support to large numbers of learners without proportionally increasing faculty workload, potentially easing strain on educational resources and addressing variability in teaching quality. Over time, this could contribute to a more uniformly trained and knowledgeable cohort of new doctors.

Specialty-specific performances can also guide students in how to use ChatGPT. Certain domains, such as surgery, infectious diseases, psychiatry, sexual health, and statistics, showed excellent performance in our analysis, perhaps because these areas are supported by well-developed knowledge bases and clearly defined guidelines. Conversely, weaker performance in GP calls for more caution, as reflected by GPT-4o’s 71% accuracy. GP spans multiple disciplines and often involves nuanced decisions about referrals and psychosocial factors. Interestingly, o1-Pro improved on most of these items, suggesting that the gap may narrow with further model development. This may indicate that in future, educators could focus teaching efforts on areas where AI is less proficient.

Our results are consistent with the growing body of evidence showing that LLMs can equal or surpass medical students on knowledge assessments. If AI systems can pass traditional exams, medical schools may shift greater emphasis to observed clinical encounters and skills assessments that AI cannot emulate. In the meantime, forward-thinking educators are considering ways to incorporate AI into teaching rather than excluding it [18]. For example, ChatGPT can generate practice quizzes or clinical scenarios for students, as noted in a student-perspective editorial, and it can also serve as a conversational study partner. The key going forward is to harness AI’s strengths, such as its vast knowledge base and constant availability, while mitigating its weaknesses [19].

### Limitations

First, although the item set was the largest analyzed to date, it may not fully capture the breadth of the official exam. The actual exam’s standard-setting and item formats may also differ slightly from those of the item bank. Second, our limited inclusion of image-based items meant we did not fully assess the multimodal capabilities of newer ChatGPT versions, which can now process images at a higher level. Third, we compared only 2 ChatGPT models. Other LLMs, such as Google’s Bard or open-source alternatives, were not evaluated. Prior studies suggest GPT currently outperforms most other publicly available LLMs on medical items, so our focus on GPT variants was reasonable; however, similar evaluations will be valuable as competitors improve. Fourth, we did not involve students directly, so comparisons with human performance remain indirect. Fifth, although McNemar’s test demonstrated a significant difference between models, we did not adjust for multiple comparisons across specialties. Some specialties contained very few items, such as social and population health with only one, so percentage differences should not be over-interpreted. Our specialty analysis was exploratory and intended to generate hypotheses that would require confirmation with larger samples. Finally, the performance of ChatGPT on the UKMLA in this study cannot be extrapolated to other examinations, such as the USMLE, because each assessment differs in structure, content, and reasoning style. For example, the UKMLA places particular emphasis on UK-specific guidelines and public health systems.

### Suggestion for further studies

Future research should include larger samples of image-based items, enabling performance comparisons across specialties and across LLMs. More detailed specialty-specific analyses could help identify precise areas of weakness within lower-scoring domains. In addition, comparative evaluations involving other LLMs (e.g., Google Gemini/Bard, Claude) with transparent version histories would allow progress to be tracked over time.

### Conclusion

This study demonstrates that ChatGPT has promising potential as a revision tool for UK medical students preparing for the UKMLA, delivering high accuracy across a wide range of clinical items. While it should not replace traditional study methods or expert-led teaching, its adaptability and accessibility may strengthen medical education by identifying knowledge gaps and reinforcing learning.

## Figures and Tables

**Figure f4-jeehp-22-30:**
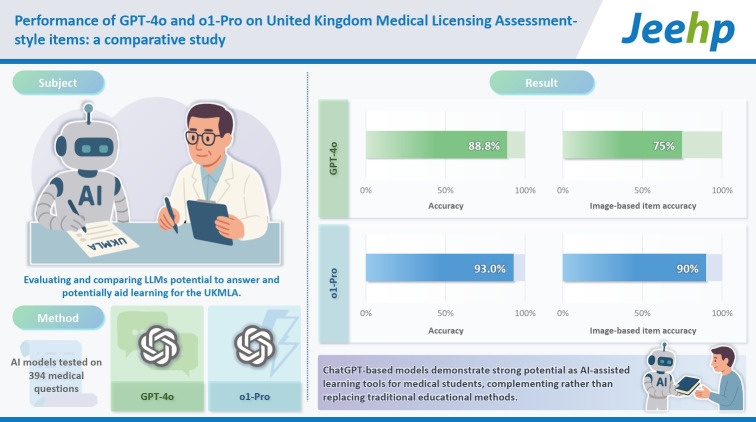


**Table 1.** Accuracy of GPT-4o and o1-Pro by specialty category on the UK Medical Licensing Assessment-style question set

Specialty	No. of questions	GPT-4o accuracy, % (no./total)	O1Pro accuracy, % (no./total)	Difference (+/– %)
Acute & emergency medicine	26	92.3 (24/26)	96.2 (25/26)	+3.9
Breast	11	81.8 (9/11)	90.9 (10/11)	+9.1
Cancer	12	75.0 (9/12)	83.3 (10/12)	+8.3
**Cardiology**	**24**	91.7 (22/24)	87.5 (21/24)	–4.2
**Dermatology**	**11**	**72.7 (8/11)**	**90.9 (10/11)**	**+18.2**
Endocrine & metabolic	18	94.4 (17/18)	94.4 (17/18)	0.0
ENT	14	92.9 (13/14)	92.9 (13/14)	0.0
Ethics & law	10	100 (10/10)	100.0 (10/10)	0.0
Gastroenterology	22	90.9 (20/22)	95.5 (21/22)	+4.6
Geriatrics (medicine of older adults)	8	87.5 (7/8)	87.5 (7/8)	0.0
**General practice**	**14**	**71.4 (10/14)**	**85.7 (12/14)**	**+14.3**
**Gynecology & obstetrics**	**19**	**78.9 (15/19)**	**89.5 (17/19)**	**+10.5**
Hematology	11	81.8 (9/11)	90.9 (10/11)	+9.1
Infectious diseases	11	100.0 (11/11)	100.0 (11/11)	0.0
Musculoskeletal	14	85.7 (12/14)	85.7 (12/14)	0.0
Neurology	17	88.2 (15/17)	94.1 (15/17)	+5.9
**Ophthalmology**	**10**	**80.0 (8/10)**	**90.0 (9/10)**	**+10.0**
Pediatrics	19	84.2 (16/19)	89.5 (17/19)	+5.3
Palliative medicine	7	100.0 (7/7)	100.0 (7/7)	0.0
Perioperative/anesthesia	8	100.0 (8/8)	100.0 (8/8)	0.0
Psychiatry	13	100.0 (13/13)	100.0 (13/13)	0.0
Renal & urology	19	89.5 (17/19)	89.5 (17/19)	0.0
**Respiratory**	**11**	**81.8 (9/11)**	**100.0 (11/11)**	**+18.2**
Rheumatology	11	81.8 (9/11)	90.9 (10/11)	+9.1
Sexual health	10	100.0 (10/10)	100.0 (10/10)	0.0
Social and population health	1	100.0 (1/1)	100.0 (1/1)	0.0
Statistics	9	100.0 (9/9)	100.0 (9/9)	0.0
Surgery	14	100.0 (14/14)	100.0 (14/14)	0.0
**Total**	**374**	**88.8**	**93.0**	

Both models achieved high scores across specialties, with o1-Pro generally matching or exceeding GPT-4o. The largest differences are highlighted in bold.

## Footnotes

**Data availability**
Data files are available from https://doi.org/10.7910/DVN/KVAMLJ Dataset 1. Responses of GPT4o and o1-Pro to the 394 United Kingdom Medical Licensing Assessment-style single-best answer items.

Dataset 2. Responses of GPT4o and o1-Pro to the 20 United Kingdom Medical Licensing Assessment-style image-based items.

## Supplementary materials

Supplement 1. Python code for the analysis of Datasets 1 and 2.

Supplement 2. Audio recording of the abstract.

## References

[1] Mir MM, Mir GM, Raina NT, Mir SM, Mir SM, Miskeen E, Alharthi MH, Alamri MM (2023). Application of artificial intelligence in medical education: current scenario and future perspectives. J Adv Med Educ Prof.

[2] Collins C, Dennehy D, Conboy K, Mikalef P (2021). Artificial intelligence in information systems research: a systematic literature review and research agenda. Int J Inf Manag.

[3] Sarker IH (2022). AI-based modeling: techniques, applications and research issues towards automation, intelligent and smart systems. SN Comput Sci.

[4] Lai UH, Wu KS, Hsu TY, Kan JK (2023). Evaluating the performance of ChatGPT-4 on the United Kingdom Medical Licensing Assessment. Front Med (Lausanne).

[5] van Dis EA, Bollen J, Zuidema W, van Rooij R, Bockting CL (2023). ChatGPT: five priorities for research. Nature.

[6] Knoedler L, Knoedler S, Hoch CC, Prantl L, Frank K, Soiderer L, Cotofana S, Dorafshar AH, Schenck T, Vollbach F, Sofo G, Alfertshofer M (2024). In-depth analysis of ChatGPT’s performance based on specific signaling words and phrases in the question stem of 2377 USMLE step 1 style questions. Sci Rep.

[7] Brin D, Sorin V, Vaid A, Soroush A, Glicksberg BS, Charney AW, Nadkarni G, Klang E (2023). Comparing ChatGPT and GPT-4 performance in USMLE soft skill assessments. Sci Rep.

[8] Sun L, Yin C, Xu Q, Zhao W (2023). Artificial intelligence for healthcare and medical education: a systematic review. Am J Transl Res.

[9] Casals-Farre O, Baskaran R, Singh A, Kaur H, Ul Hoque T, de Almeida A, Coffey M, Hassoulas A (2025). Assessing ChatGPT 4.0’s capabilities in the United Kingdom Medical Licensing Examination (UKMLA): a robust categorical analysis. Sci Rep.

[10] Saowaprut P, Wabina RS, Yang J, Siriwat L (2025). Performance of large language models on Thailand’s national medical licensing examination: a cross-sectional study. J Educ Eval Health Prof.

[11] Torres-Zegarra BC, Rios-Garcia W, Nana-Cordova AM, Arteaga-Cisneros KF, Chalco XCB, Ordonez MA, Rios CJ, Godoy CA, Quezada KL, Gutierrez-Arratia JD, Flores-Cohaila JA (2023). Performance of ChatGPT, Bard, Claude, and Bing on the Peruvian National Licensing Medical Examination: a cross-sectional study. J Educ Eval Health Prof.

[12] Fowler T, Pullen S, Birkett L (2024). Performance of ChatGPT and Bard on the official part 1 FRCOphth practice questions. Br J Ophthalmol.

[13] Chen R, Fettel KD, Nguyen DH, Nambudiri VE (2025). Evaluating the performance of ChatGPT on dermatology board-style exams: a meta-analysis of text-based and image-based question accuracy. J Am Acad Dermatol.

[14] Jang D, Yun T, Lee CY, Kwon YK, Kim CE (2025). Correction: GPT-4 can pass the Korean National Licensing Examination for Korean Medicine Doctors. PLOS Digit Health.

[15] Kung TH, Cheatham M, Medenilla A, Sillos C, De Leon L, Elepano C, Madriaga M, Aggabao R, Diaz-Candido G, Maningo J, Tseng V (2023). Performance of ChatGPT on USMLE: potential for AI-assisted medical education using large language models. PLOS Digit Health.

[16] Mbakwe AB, Lourentzou I, Celi LA, Mechanic OJ, Dagan A (2023). ChatGPT passing USMLE shines a spotlight on the flaws of medical education. PLOS Digit Health.

[17] Wartman SA, Combs CD (2018). Medical education must move from the information age to the age of artificial intelligence. Acad Med.

[18] Boscardin CK, Gin B, Golde PB, Hauer KE (2024). ChatGPT and generative artificial intelligence for medical education: potential impact and opportunity. Acad Med.

[19] Mohammad B, Supti T, Alzubaidi M, Shah H, Alam T, Shah Z, Househ M (2023). The pros and cons of using ChatGPT in medical education: a scoping review. Stud Health Technol Inform.

